# Analysis of Cause-Specific Mortality in Patients with Retinoblastoma

**DOI:** 10.1155/2022/2470890

**Published:** 2022-03-04

**Authors:** Lei Yang, Xiaolian Fang, Mei Jin, Jun Chen, Chengyue Zhang, Jianing Mou, Junyang Zhao, Xin Ni

**Affiliations:** ^1^Department of Otolaryngology, Head and Neck Surgery, Beijing Children's Hospital, Capital Medical University, National Center for Children's Health, Beijing 100045, China; ^2^Medical Oncology Department, Pediatric Oncology Center, Beijing Children's Hospital, Capital Medical University, National Center for Children's Health, Beijing Key Laboratory of Pediatric Hematology Ocology, Key Laboratory of Major Diseases in Children, Ministry of Education, Beijing 100045, China; ^3^Beijing Engineering Research Center of Pediatric Surgery, Engineering and Transformation Center, Beijing Children's Hospital, Capital Medical University, National Center for Children's Health, Beijing, China; ^4^Department of Ophthalmology, Beijing Children's Hospital, Capital Medical University, National Center for Children's Health, Beijing 100045, China; ^5^Pediatric Oncology Center, Beijing Children's Hospital, Capital Medical University, National Center for Children's Health, Beijing 100045, China

## Abstract

**Background:**

Retinoblastoma (RB) is a rare pediatric tumor with a relatively favorable prognosis. However, RB is associated with cause-specific mortality, some of that should be of great importance to clinicians. In this study, we summarize the characteristics of cause-specific mortality from nontumor disease in patients with RB.

**Methods:**

This retrospective case series study identified and analyzed cause-specific mortality in patients with RB. The information of cause-specific mortality of RB patients, including detailed clinical characteristics, diagnosis, treatment process, cause-specific mortality classification, and lag time, was assessed.

**Results:**

A total of 12 eligible patients were selected from 264 patients who died among 3780 patients diagnosed with RB. The cause-specific mortality rate was 4.5% for all patients with RB who died and 0.3% for all patients with RB. The main nontumor cause-specific mortalities were diseases of the nervous, circulatory, and respiratory systems, which specifically included intracranial infection, cerebral hemorrhage, paraplegia, and respiratory failure. The longest lag time was 42 days from the last chemoradiotherapy or surgery.

**Conclusion:**

Nontumor cause-specific mortality is an essential outcome of RB. Thus, intensive care and differentiation during management need to be taken seriously.

## 1. Introduction

Retinoblastoma (RB) is a rare ocular tumor. However, it is the most common intraocular tumor in childhood. It is initially caused by a tumor-suppressor mutation of *RB1* [1]. Early diagnosis and multifarious surgery or chemoradiotherapy sharply decrease the mortality rate of patients with RB. At present, comprehensive treatment of RB involves administration of intravenous chemotherapy (IVC), intraophthalmic artery chemotherapy (IAC), and periocular and intravitreal techniques [2]. IVC and IAC in particular have been found to be safe and efficient for the treatment of RB. Both therapies have been reported to gradually preserve the eye and increase the five-year survival rate of patients with RB [3, 4]. Meanwhile, a combination of IAC and intravitreal chemotherapy has higher success rates and lower complication rates [5]. However, patients with RB still have a high risk of developing subsequent primary malignancies or other systemic diseases, which could be life-threatening [6, 7].

Previous studies have shown that the cause of mortality of patients with RB is related to subsequent malignancies and other cause-specific mortality, including infections, endocrine and metabolic diseases, mental disorders, neurological diseases, circulatory diseases, respiratory diseases, and digestive system diseases (according to the International Classification of Disease (ICD) codes) [6, 8]. Subsequent malignancy has been reported to be a greater risk factor among hereditary retinoblastoma survivors and is associated with radiotherapy [9, 10]. However, the nontumor cause-specific mortality still presents a potential risk for patients with RB [1, 11]. The aim of this study was to analyze cause-specific mortality in patients with RB, especially those with other systemic nontumor diseases and summarize their clinical characteristics. We emphasized nontumor cause-specific mortality in patients with RB to raise awareness of clinical treatment and survival of RB and help decrease the mortality rate of RB.

## 2. Methods

In this retrospective case series study, we identified and analyzed cause-specific mortality in patients with RB that was initially diagnosed and treated between 2006 and 2019 at the Beijing Children's Hospital and Beijing Tongren Hospital. All procedures were performed, and written informed consent was obtained in accordance with the relevant guidelines and regulations of the Declaration of Helsinki.

We collected the detailed information of the patients with RB from electronic medical records with standardized forms of basic clinical information, detailed diagnostic and treatment processes, details of the last therapy, and detailed time, circumstances, and classification of cause of mortality. The cause mortality of patients was classified according to the ICD, and the cause-specific mortality was defined as the mortality from other systemic nontumor diseases, excluding mortality due to tumors or metastases.

All patients with RB were clinically diagnosed based on the presence of abnormal ocular symptoms, including leukocoria, vision loss, or hypopyon, and findings of detailed fundus examination, computed tomography, or magnetic resonance imaging. Clinical disease stages at the time of diagnosis and treatment period were categorized according to the standards of the International Intraocular Retinoblastoma Classification (IIRC). All patients were treated using standard treatments, including IVC or IAC, radiotherapy, and surgical enucleation.

Lag time, which is a relatively life-threatening period for RB patients, was defined as the duration from the end of the last therapy to the time of death. Diagnostic delay time was defined as the duration from the detection of the first sign to the time of diagnosis. Treatment delay time was defined as the duration from the time of diagnosis to the initiation of primary treatment. Survival time was defined as the duration from the time of diagnosis to the time of death. All causes of death were considered classified variables, whereas the time of diagnosis, therapy, and death were considered numeric variables. The average diagnostic delay time, death time, treatment delay time, survival time, and lag time were calculated and summarized.

## 3. Results

**Figure 1:**
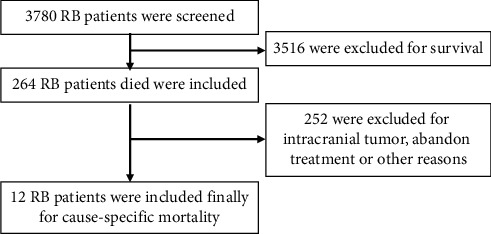
Eligibility evaluation flow diagram for 12 RB cause-specific mortality patients. A total of 3780 RB patients were initially screened. After excluding 3516 survivors, 264 RB patients who died were included. After excluding 252 patients for intracranial tumor, abandonment of treatment, or other reasons, 12 RB cause-specific mortality patients were finally included.

A total of 12 eligible nontumor cause-specific mortality patients were selected from 264 patients who died among 3780 patients diagnosed with RB and included in this study. The cause-specific mortality rate was 4.5% for all patients who died and 0.3% for all patients with RB. The eligibility evaluation flow diagram (Figure 1) clearly showed the 12 cause-specific mortality RB patients selected for participating in this study.

The clinical information of the selected 12 patients with RB is displayed in Table 1. The 12 patients were equally distributed in terms of sex and laterality of diagnoses. Leukocoria was the main initial symptom, followed by vision loss and hypopyon. Most patients visited our hospital for the first time, two patients were referred to Beijing Children's Hospital, and one patient was treated in another hospital after consultation in our hospital.

Table 2 shows the specific clinical information of 12 cause-specific mortality patients with RB. Regarding RB stages at the time of diagnosis, six cases of unilateral RB were in stages D, E, and E+ at the time of diagnosis, whereas six cases of bilateral RB were in stages B/E, C/D, D/D, ?(unclear)/E, and D/E+. Regarding RB stages at the last evaluation, the six cases of unilateral RB were in stable stage E, chemotherapy duration stages D and E, and enucleation stages E, E, and E+, whereas the six cases of bilateral RB were in the stable/enucleation and chemotherapy duration stages.

Regarding the therapy processes of the patients, nine patients in group D or E initially underwent IVC therapy, carboplatin, etoposide, vincristine (CEV), or carboplatin, teniposide, vincristine (CTV) on an average of 2.7 (1–7) times and subsequently accepted enucleation, radiotherapy, or intraocular laser therapy; one patient in group D with bilateral low vision was initially treated with IAC two times; and two patients in group E initially underwent enucleation and subsequently accepted several sessions of IVC. However, although five of these patients (lines 1, 2, 6, 7, and 11 in Table 2) who underwent enucleation or systematic IVC were evaluated as stable and were considered to should survival, the cause-specific mortality still happened after their last regular treatment.

We summarized the nontumor cause-specific mortality classification, last therapy, and lag time of the 12 patients with RB (Table 3). The cause-specific mortality of the included patients was divided into mortality from diseases of the nervous, circulatory, and respiratory systems, specifically intracranial infection (ICD-G06.003), paraplegia (ICD-G82.205), cerebral hemorrhage (ICD-I61.902), and respiratory failure (ICD-I96.051). Seven patients died of nervous system disease: five of the patients had intracranial infections, whereas two had paraplegia an average of 7.8 and 20.5 days after their last chemotherapy or radiotherapy session. Three patients died of cerebral hemorrhage an average of 15 days after their last chemotherapy session. Two patients died of respiratory failure an average of 6.5 days after their last chemotherapy session or surgery.

The average diagnostic age of the 12 patients with RB was 17.7 (1.2–97.2) months. The average diagnostic delay time was 1.2 (0.2–4.9) months, and the average treatment delay time was 2.1 (0.3–12.1) months. The average age of the patients at the time of death was 23.9 (1.5–105.8) months, whereas their average survival time was 6.2 (0.3–27.1) months (Table 4).

## 4. Discussion

In this paper, we focused on the cause-specific mortality of 12 patients with RB and summarized their details of basic clinical information, diagnoses, treatments, and mortality. We specifically analyzed cause-specific mortality from other systemic nontumor diseases, including diseases of the nervous, circulatory, and respiratory systems, especially intracranial infection, paraplegia, cerebral hemorrhage, and respiratory failure.

In this study, a total of 12 cause-specific mortality patients with RB from other systematic nontumor diseases were selected from 264 patients who died in a cohort of 3780 patients with RB. Our results are partly consistent with those of some previous RB follow-up research. Temming et al. [12] reported cause-specific mortality due to nontumor disease in 2 out of 43 patients who died in a cohort of 633 patients with RB in Germany. Yu et al. [6] reported cause-specific mortality from nontumor disease in 39 out of 211 who died during a one-year follow-up study in the United States (US). Waddell et al. [13] revealed that among 108 deaths recorded in a cohort of 270 patients with RB in Uganda, one case of leucopenia after chemotherapy may be due to infection. Broaddus et al. [3] identified three cases of septicemia mortality among 63 patients in the SEER database who died. Kleinerman et al. [8] found 98 cases of cause-specific mortality from nontumor disease among 690 deaths recorded in a long-term follow-up from 1914 to 2016 in the US. Marees et al. [14] summarized 99 cases of cause-specific mortality from nontumor disease, mostly circulatory or cardiovascular disease, among 332 patients during a long-term follow-up from 1862 to 2005 in the Netherlands. The infectious mortality rate for patients with RB recorded in these previous studies ranged from 0.7% to 4.8% [3, 6, 8, 12, 13], whereas the cerebral hemorrhage mortality rate was 1.5% [14]. The infection mortality rate in our present study was 1.9% and the cerebral hemorrhage mortality rate was 1.1%, whereas the total nontumor cause-specific mortality rate was 4.5% and 0.3% for all patients who died and all patients with RB, respectively.

Cause-specific mortality due to nontumor disease occurs during the course of RB. Therefore, increased attention should be paid to the occurrence of nontumor diseases in cases of RB. In this study, we determined the lag time from the end of the last therapy to death, which is a vital and risky period for patients with RB. During this period, doctors need to pay attention to patient care from the aspects of chemotherapy, radiotherapy, and surgical management. Regarding the perioperative period of regular chemotherapy or enucleation, close monitoring of patients with RB for at least 19 days is essential to get through the lag time. For radiotherapy, we suggest strengthening intensive care for more than 40 days after the therapy to expedite the effects of the treatment and reduce possible damage due to radiation [15, 16].

The most common systemic complications of IVC and IAC are neutropenia, transient fever, and nausea/vomiting, whereas the most common ocular complication is retinal detachment [17–20]. Researchers have proved that increased frequency of chemotherapy, drug dose accumulation, and previously failed treatment are independent risk factors for ocular motility complications during the treatment of RB [11, 21], which may be hazardous to patients. There are still some accidents and complications that occur during the common treatment of RB that alert clinicians to differentiate the preaccident mortality symptoms. In the present study, all cases of intracranial infection and cerebral hemorrhage mortality occurred after specific IVC or IAC sessions performed for approximately two weeks; other symptoms such as paraplegia and respiratory failure were also observed. These specific symptoms indicate that more attention should be paid to the condition of patients with RB during or after treatment. In addition, physicians should be alert to the occurrence of cause-specific mortality and consider specific perioperative management, especially for patients with stage D/E disease or those who underwent multiple chemoradiotherapy sessions.

Tumor therapy has advanced over the years; however, the safety and efficiency of novel and traditional therapies still need to be evaluated. Although the new method of IAC is as efficient as traditional IVC or enucleation in some specific cases of RB [20], vascular toxicity and local complications are curtailed with IAC. However, the proper dose of IAC is unpredictable for complex and variable conditions [22]. An increasing number of novel therapies, such as focal lasers [23] and intravitreal chemotherapy [24] have emerged as therapeutic options for RB. Furthermore, researchers have found that a white blood cell count lower than 1 × 10^9^ strongly increases the risk of infection. Some clinicians administer recombinant human granulocyte colony-stimulating factor (rhG-CSF) on the fourth day after regular chemotherapy, which may provide a protective benefit against infection-related accidents. However, it has been suggested that rhG-CSF be used only when anticipating long-term agranulocytosis. Preventing cause-specific mortality is essential for patients with RB who are judged to have chances of survival. In the present study, 5 of the 12 included patients were in the stable stage at the last evaluation and were considered to should survival after appropriate treatment (lines 1, 2, 6, 7, and 11 in Table 2). As the multifarious treatment advanced, we should still take the original and intensive care for RB therapy period and differentiate the severe symptom avoiding the cause-specific mortality from nontumor disease.

In this study, the average ages of the 12 patients at the time of diagnosis and death were relatively young. In addition, delays in diagnosis and treatment were observed in this study (Table 4). The average survival time (6 months) of the included patients was much shorter than that of the overall patients with RB. Proper diagnosis and appropriate timing and planning of treatment may also play essential roles in the prognosis of RB and the prevention of nontumor cause-specific mortality [25, 26].

One limitation of the present study was information bias, which selected cases based on the hospital's electronic medical record system in the regional center in China and limited its generalization. Besides, the present study does not assess causal relationship for cause-specific mortality in RB patients, and thus, more prospective collaboration in multicenter studies with longer follow-up is needed to generate higher-quality evidence in the future.

## 5. Conclusion

RB could have a good prognosis after comprehensive treatment; however, cause-specific mortality from nontumor diseases still needs to be considered. The most common cause-specific mortalities in present cases of RB are nervous, circulatory, and respiratory system diseases. Intensive care and differentiation are essential during the lag time from the last chemoradiotherapy or surgery.

## Figures and Tables

**Table 1:** Characteristics of 12 RB cause-specific mortality patients.

Characteristics	Cause-specific mortality (*N* = 12)
Diagnosis date	2007–2019
Sex	
Male	6 (50.0%)
Female	6 (50.0%)
Laterality	
Unilateral	6 (50.0%)
Bilateral	6 (50.0%)
Initial symptom	
Leukocoria	10 (83.3%)
Vision loss	1 (8.3%)
Hypopyon	1 (8.3%)
Patients source	
Primary hospital	9 (75.0%)
Referral	2 (16.7%)
Consultant	1 (8.3%)
Family history	
No	11 (91.7%)
Yes	1 (8.3%)
Initial treatment	
IVC	9 (75.0%)
IAC	1 (8.3%)
Enucleation	2 (16.7%)
Last therapy	
Chemotherapy	10 (83.3%)
Surgery	1 (8.3%)
Radiotherapy	1 (8.3%)
Direct death reason	
Intracranial infection	5 (41.7%)
Cerebral hemorrhage	3 (25.0%)
Respiratory failure	2 (16.7%)
Paraplegia	2 (16.7%)

**Table 2:** Clinical features of 12 RB cause-specific mortality patients.

Patient no. (laterality)	Gender	Age of Dx (months)	IS	Origin	FH	Diagnostic stage (IIRC)	Initial treatment	Treatment after diagnosis	Metastasis	Survival time (months)	Age of death (months)	Last therapy	Direct death reason	Last therapy to death (days)	Last evaluation	Addition
1 (B)	M	25.3	Leukocoria	Referral	No	?/E	En	En, CEV3	No	6.5	31.8	Surgery	Respiratory failure	6	En/S	Should survival
2 (B)	M	2.2	Leukocoria	Primary	No	B/E	IVC	CEV3, laser	No	5.4	7.7	Chemo	Cerebral hemorrhage	13	S/En	Should survival
3 (B)	M	4.4	Leukocoria	Primary	No	D/C	IVC	CEV2	No	1.8	6.2	Chemo	Cerebral hemorrhage	13	T/T	
4 (U)	F	15.3	Leukocoria	Primary	No	E+	IVC	CEV4, En, Radio	Yes	7.9	23.2	Radio	Paraplegia	40	En	
5 (B)	F	15.1	Low vision	Primary	Father (RB+)	D/D	IAC	IAC2	No	1.4	16.5	Chemo	Intracranial infection	8	T/T	
6 (U)	F	97.2	Hypopyon	Referral	No	E	IVC	CTV1	No	8.6	105.8	Chemo	Cerebral hemorrhage	19	S	Should survival
7 (U)	M	4.5	Leukocoria	Primary	No	E	En	En, CTV2	No	1.2	5.7	Chemo	Intracranial infection	7	En	Should survival
8 (U)	F	1.2	Leukocoria	Primary	No	E	IVC	CEV1	No	0.3	1.5	Chemo	Respiratory failure	7	T	
9 (U)	M	4.5	Leukocoria	Consulting	No	D	IVC	CEV1	No	0.6	5.1	Chemo	Intracranial infection	12	T	
10 (B)	M	22.9	Leukocoria	Primary	No	D/E+	IVC	CEV1, CTV4, intrathecal	Yes (CSF+)	11.5	34.4	Chemo	Intracranial infection	2	T/T	
11 (U)	F	1.7	Leukocoria	Primary	No	E	IVC	CTV2, En, CTV1	No	2	3.8	Chemo	Paraplegia	1	En	Should survival
12 (B)	F	17.7	Leukocoria	Primary	No	C/D	IVC	CEV7, IAC11, En CEV6, intrathecal	No	27.1	44.7	Chemo	Intracranial infection	10	T/En	

B = bilateral; U = unilateral; S = stable; En = enucleation; T = tumor; M = male; F = female; Dx = diagnosis; IS = initial symptom; FH = family history;? = unclear; CEV = carboplatin, etoposide, vincristine; CTV = carboplatin, teniposide, vincristine; Chemo = chemotherapy; Radio = radiotherapy.

**Table 3:** Nontumor cause-specific mortality classification, last therapy, and lag time of 12 RB patients.

Mortality causes	ICD-10	Total number	Last therapy	Average lag time (day, min-max)
Nervous system				
Intracranial infection	G06.003	5	Chemo	7.8 (2–12)
Paraplegia	G82.205	2	Chemo/radio	20.5 (1–40)
Circulatory system				
Cerebral hemorrhage	I61.902	3	Chemo	15 (13–19)
Respiratory system				
Respiratory failure	J96.051	2	Chemo/surgery	6.5 (6-7)

**Table 4:** Clinical average time information of 12 RB cause-specific mortality patients.

Age and time	Average month (min–max)
Diagnostic age	17.7 (1.2–97.2)
Death age	23.9 (1.5–105.8)
Diagnostic delay time	1.2 (0.2–4.9)
Treatment delay time	2.1 (0.3–12.1)
Survival time	6.2 (0.3–27.1)

## Data Availability

The clinical information data used to support the findings of this study are available from the corresponding author upon request.
